# Synthesis and Characterization of a Novel Borazine-Type UV Photo-Induced Polymerization of Ceramic Precursors

**DOI:** 10.3390/molecules21060801

**Published:** 2016-06-21

**Authors:** Dan Wei, Lixin Chen, Tingting Xu, Weiqi He, Yi Wang

**Affiliations:** Department of Applied Chemistry, School of Science, Northwestern Polytechnical University, Xi’an 710129, Shaanxi, China; 1990weidan@sina.com (D.W.); tingtingxu@nwpu.edu.cn (T.X.); vikeyhe@sina.com (W.H.); wangyi4712@sina.com (Y.W.)

**Keywords:** single-source precursor, UV curing, six-membered borazine rings, X-ray methods

## Abstract

A preceramic polymer of B,B′,B′′-(dimethyl)ethyl-acrylate-silyloxyethyl-borazine was synthesized by three steps from a molecular single-source precursor and characterized by Fourier transform infrared (FTIR) and nuclear magnetic resonance (NMR) spectrometry. Six-member borazine rings and acrylate groups were effectively introduced into the preceramic polymer to activate UV photo-induced polymerization. Photo-Differential Scanning Calorimetry (Photo-DSC) and real-time FTIR techniques were adapted to investigate the photo-polymerization process. The results revealed that the borazine derivative exhibited dramatic activity by UV polymerization, the double-bond conversion of which reached a maximum in 40 s. Furthermore, the properties of the pyrogenetic products were studied by scanning electron microscopy (SEM) and X-ray diffraction (XRD), which proved the ceramic annealed at 1100 °C retained the amorphous phase.

## 1. Introduction

Precursor-derived quaternary Si/B/C/N random networks consisting of the elements silicon, carbon, boron and nitrogen, exhibit remarkably high thermal, chemical, and mechanical stability in oxygen free atmospheres, even at temperatures up to 2000–2200 °C [1,2,3,4,5,6]. Si/B/C/N can be prepared by the powder-sintering method, the chemical-vapor-deposition (CVD) method, the chemical-vapor-infiltration (CVI) method and as polymer-derived ceramics (PDCs). Compared with traditional technologies to prepare ceramics, the PDC route uses monomers or polymers to fabricate preceramic precursor, and the final ceramic products are obtained through processing and pyrolysis of the preceramic precursors [7]. The PDC method has many advantages, for example, the preceramic polymers can be processed or shaped using usual polymer forming techniques such as resin transfer molding (RTM), polymer infiltration pyrolysis (PIP), injection molding or solvent coating [1,5,8,9]. Meanwhile, the composition and structure of the ceramics can also be adjusted by rational structural design of the precursor molecules. PDCs have drawn a lot of attention recently due to their unique mechanical and chemical properties and their potential applications in the area of high-temperature-resistant materials, hard materials, chemical engineering, or functional materials, *etc.* [1,8,10]. In the PDC method, the polymerization stage plays a crucial role in determining the final properties of the resulting ceramic materials. Single molecules can be polymerized via chemical, thermal, photo- induced and other approaches. Conventional chemical polymerization processes need the presence of reactive agents such as methylamine [11], ammonia, thionyl chloride, nitrogen dioxide, nitric oxide, halogenated or unsaturated hydrocarbon, *etc.* These processes produce small molecular by-products which need to be further treated and can be very difficult to remove [12]. Photo-induced polymerization strategies can be carried out under ambient conditions, and can also be applied in solvent-free systems. In addition, a wide variety of monomeric structures are available and can control the extent of polymerization [13]. Nason *et al.* discussed a clear relationship between the traveling front velocity and monomer structure using the photo-DSC method [14], Chiou studied the UV curable process of the thiol-ene system by real-time FTIR spectroscopy [15], and Kong also determined the UV curing behavior of (meth)acrylic pendant groups in polyorganosilazane precursors via FTIR [16,17]. It demonstrated that real-time FTIR spectroscopy offers a powerful approach to monitor photo-sensitive groups in the polymerization process of preceramic polymers during UV curing. The conversion of unsaturated bonds at any time can be calculated from the first integration of the unreacted peak at the starting point of the experiment (A_0_) and the integrated area of the peak at time t (A_t_). The conversion(x) of unsaturated bond at time t can be determined as follows: x(t) = 1−A_t_/A_0_ [15,16,17,18,19].

Although the photo-induced polymerization methods to prepare preceramic polymers have been investigated, there are few reports about the final ceramic products. Herein, we have introduced acrylate as a photo-sensitive functional group into the backbone of preceramic polymers to enable UV-curing [1,2,3]. A new single source precursor with acrylate groups was synthesized, and its structure was characterized by NMR and FTIR spectroscopy. Photo-DSC and real-time FTIR techniques have been used to study the UV-curing properties and subsequent changes in chemistry during curing, respectively. In addition, the new Si/B/C/N ceramic has been studied by SEM and XRD.

## 2. Results and Discussion 

### 2.1. Characterization of a-CSEB Molecule

**Figure 1:**
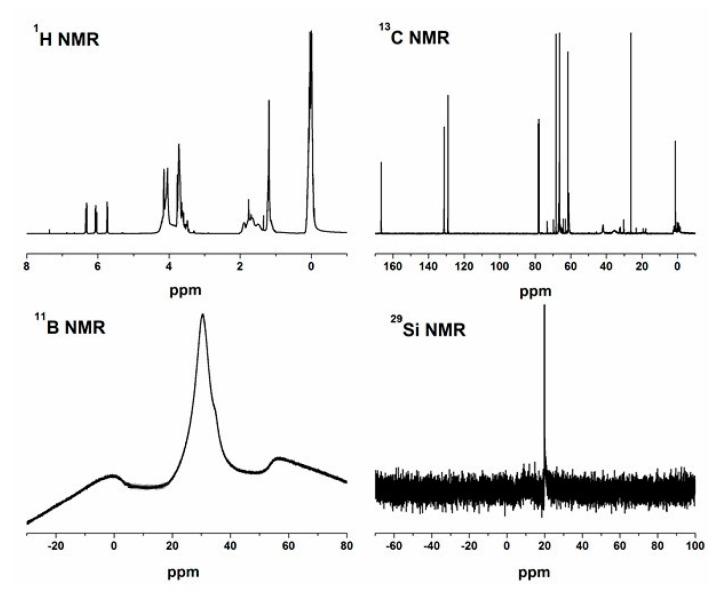
The NMR spectra of the a-CSEB molecule.

The NMR spectra of a-CSEB molecule are shown in Figure 1, as well as the NMR spectra data of a-CSEB molecule are summarized in Table 1. The ^1^H-NMR spectrum displays three signals at 6.33, 5.72 and 6.02 ppm which are attributed to –CH=C**H**_2_ and –C**H**=CH_2_ protons. The signal at about 1.75 ppm can be attributed to –C**H**–CH_3_ [20] units, and the signal of –CH–C**H**_3_ protons is observed at 1.18 ppm, while the resonance singlet signal for the N**H** proton atoms of the borazine rings appear at 4.0 ppm. In the ^13^C-NMR spectrum, intense signals at 129.03 and 131.16 ppm may originate from –**C**H=CH_2_ and –CH=**C**H_2_ carbon atoms. The sharp singlet at 166.53 ppm can be assigned to **C**=O. The signal at 26.19 ppm is assigned to –CH–**C**H_3_ as well as the peak at 68.36 ppm can be attributed to the –**C**H–CH_3_ units. The ^29^Si-NMR spectrum shows the expected signal at 19.93 ppm which is attributed to the –C–**Si**(CH_3_)_2_–O– unit [20]. In the ^11^B-NMR spectrum, a broad signal related to **B**_3_N_3_ is present at 30.00 ppm [20], which indicates the molecules have six-membered borazine rings.

**Figure 2:**
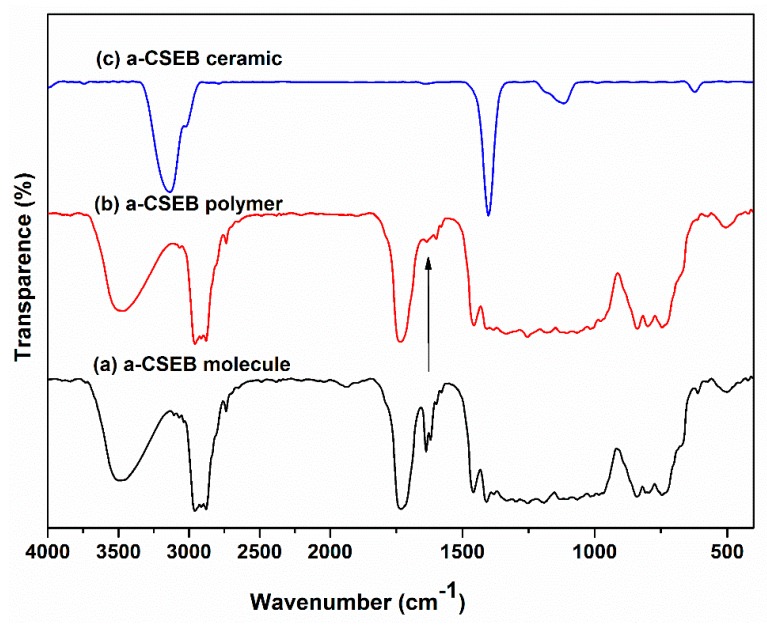
FT-IR spectra of (**a**) a-CSEB molecule; (**b**) polymer derived from a-CSEB; (**c**) ceramic derived from a-CSEB.

The FTIR spectrum of a-CSEB molecule is shown in Figure 2a. The B_3_N_3_ group stretching vibrations appear at 1462 cm^−1^ and 840 cm^−1^ [21]. Besides, the strong stretching vibration of the NH group appears at 3494 cm^−1^, while the bending vibration of the NH group is seen at 1069 cm^−1^. The C=O stretch absorption appears at 1735 cm^−1^, while the C=C group vibrations are at 1640 and 1610 cm^−1^. Likewise, a strong absorption appears at 1002 cm^−1^, attributed to Si–O bond stretching vibration. The corresponding assignments of the functional groups have been summarized in Table 2.

The FTIR and NMR results indicate that the molecule derived from CSEB shows evidence for the formation of B_3_N_3_ as six-membered borazine ring units. The resonance singlet signal for the N**H** proton atoms of borazine rings at 4.0 ppm in the ^1^H-NMR spectrum and a broad signal related to **B**_3_N_3_ is present at 30.00 ppm in the ^11^B-NMR spectrum, as well as B_3_N_3_ group stretching vibrations appear at 1462 cm^−1^ and 840 cm^−1^, along with a strong stretching vibration of the NH group at 3494 cm^−1^ and the bending vibrations of the NH group at 1069 cm^−1^, which all indicate that the molecules have six-membered borazine rings. Moreover, signals attributed to –CH=CH_2_ are visible in the ^1^H-NMR and ^13^C-NMR spectra, as well as a signal at 19.93 ppm which is attributed to the –C–**Si**(CH_3_)_2_–O– unit in the ^29^Si-NMR spectrum. Besides, C=C group vibrations appear in the FT-IR spectra at 1640 and 1610 cm^−1^, in addition to a strong absorption at 1002 cm^−1^ that is attributed to Si–O bond stretching vibration. In one word, the characterization data show that acrylate units with high photosensitivity have been successfully introduced into the molecular structure of a-CSEB which can be UV-curable. 

### 2.2. UV Polymerization Properties

**Figure 3:**
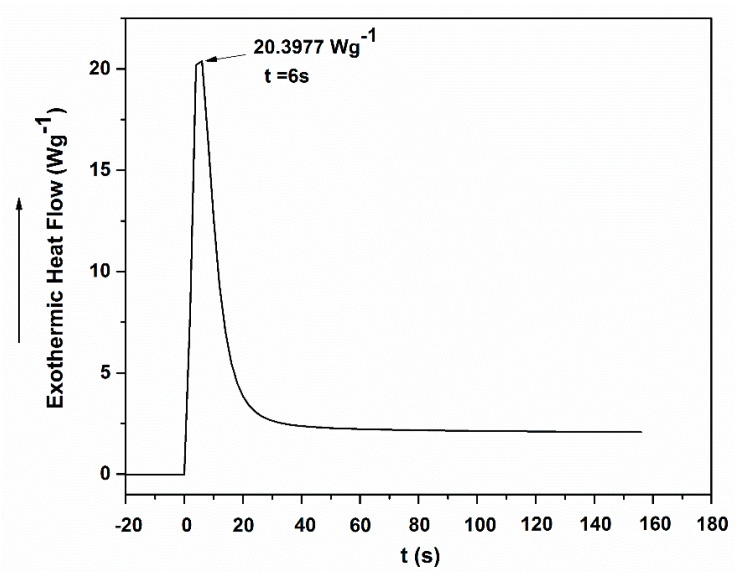
Photo-DSC curve of a-CSEB.

Photo-DSC was used to measure the photopolymerization rate of a-CSEB polymer at 25 °C. The concentration of initiator, light intensity, kind of initiator, temperature and reaction environment all affect the light curing process. In a typical experiment, the UV photo-induced polymerization was carried out using 5% benzoin as photo initiator, which was added to a-CSEB, and the resulting mixture was irradiated under a light intensity of 110 mW/cm^2^ at 25 °C. The light intensity was measured via a blackbody absorber. Parameters obtained from the photo-DSC include the polymerization exotherm, the peak exotherm maximum and the time to attain the peak maximum. As shown in Figure 3, the exothermic phenomena are very obvious, and one exothermic curing peak of 21.03 W/g is found at 6 s.

Integrating the exothermic peak demonstrates that the system curing reaction heat is 234.78 J/g, indicating that above all, a-CSEB can undergo UV photoinduced polymerization and display excellent photochemical reaction performance. FTIR experiments were also conducted to validate qualitatively the results determined from the differential scanning calorimeter analysis. Further researches on important factors for rapid UV-curable preceramic polymers in the future like initiator concentration, light intensity, atmosphere and temperature are still underway and will be report in due course.

**Figure 4:**
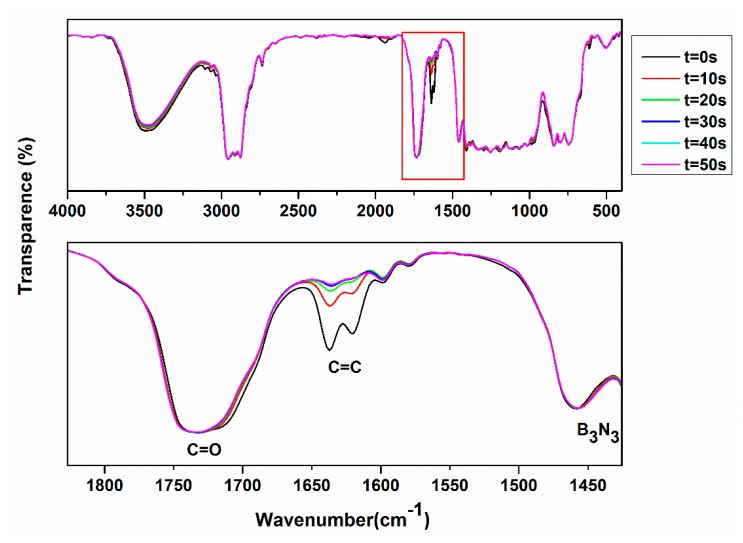
Real-time FTIR spectra of a-CSEB.

The infrared spectroscopy technique has proved to be a suitable method to monitor UV curing chemical changes of a-CSEB. During the curing progress the changes in peak area of the signal C=C at about 1637 cm^−1^, the C=O stretching vibration (1735 cm^−1^) and the peak of B_3_N_3_ group (1462 cm^−1^) [21] that did not change during the course of the reaction can be detected and used as the internal standards. In our study, the external conditions like initiator concentration, light intensity, temperature are the same as in the photo-DSC experiments. The infrared spectra of a-CSEB for six different UV exposure times are shown in Figure 4, where the lower part is a partial view shown in the red box of the upper part in the figure. It is indicated that the C=C peak area decreases with the increase in irradiation time. When the irradiation time is more than 40 s, the peak of the double bond nearly disappears. The phenomenon is due to the fact that the more the functional groups react the more the peaks gradually transform during the UV curing progress. At the end of the process, the peaks areas almost do not change since most of double bonds already reacted to form a cross-linked network. 

**Figure 5:**
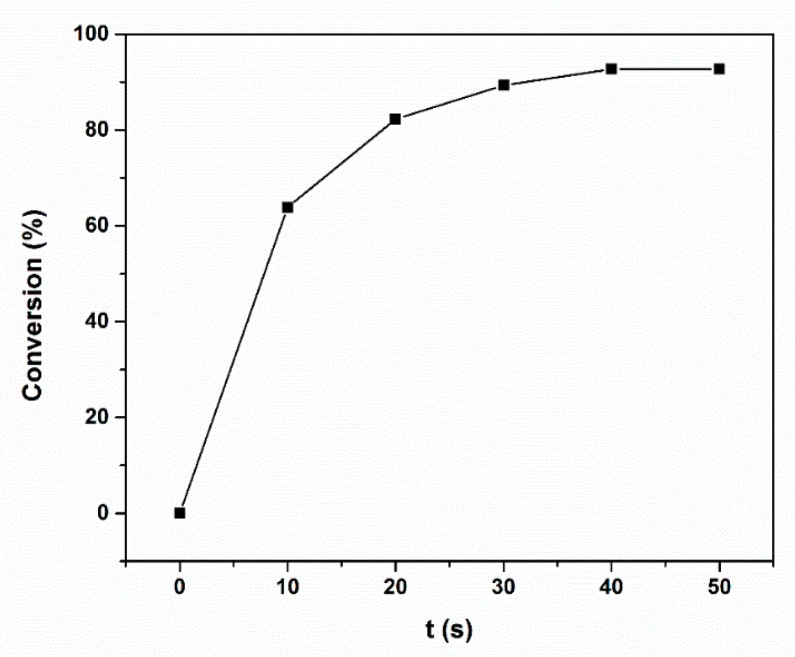
The rate of double bond conversion.

The rate of double bond conversion at different times during the UV curing process is shown in Figure 5. The peak area integrations reflect that the conversion of unsaturated bonds in the a-CSEB molecules is increasing with the increasing irradiation time. When the UV exposure time is 40 s, the conversion rate reaches a maximum value of 92.75%. It is thus indicated that UV curing can achieve a very high degree of conversion in a short period of time. One possible factor that could be light scattering causes UV radiation to penetrate deeper and thus increase the degree of conversion in the bulk medium. Furthermore, as more molecules joined the network, the final conversion increases.

According to the photo-DSC curve and real-time FTIR spectra, the a-CSEB molecule has outstanding UV polymerization properties which can achieve a very high conversion of double bonds in a very short period of time.

### 2.3. Characterization of the a-CSEB Polymer

In these experiments the photo-initiator is similarly 5% benzoin, and light intensity is 110 mW/cm^2^. Under UV light irradiation for 5 min, a UV-cured a-CSEB polymer is obtained. The NMR spectra of a-CSEB polymer (Table 3) derived from the a-CSEB molecules illustrates two important conclusions [21]. Firstly, the ^13^C-NMR and ^1^H-NMR spectra confirm that the C=C bonds are transformed into C−C bonds during the UV-induced polymerization process on the basis of a free radical curing mechanism, which demonstrates that the small a-CSEB molecules successfully polymerize into macromolecules. Secondly, according to the ^11^B-NMR results, boron atoms still exist in the six membered borazine ring covalent bonded with two nitrogen atoms and one carbon atom. In comparison with a-CSEB molecule, the FTIR curve (Figure 2b) and data (Table 2) can analyze to validate qualitatively the NMR results. Comparing to the two FTIR spectra, both show similar stretching and deformation bands except for the missing stretching of C=C at about 1640 cm^−1^ and 1410 cm^−1^, so the results are as expected.

### 2.4. Pyrolysis of the a-CSEB Polymer

**Figure 6:**
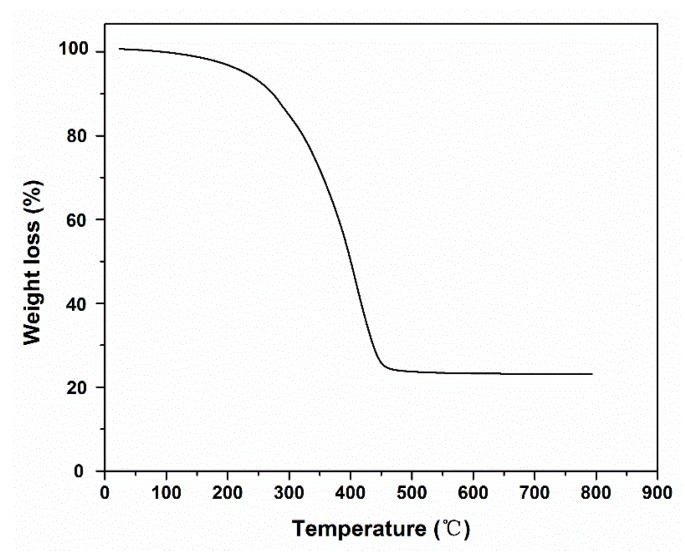
TG curve of a-CSEB polymer.

The weight change of a-CSEB polymer was measured by TG upon heating up to 800 °C in a flowing N_2_ atmosphere at 10 °C/min. As shown in Figure 6, most of the weight loss occurs between 100 °C and 450 °C. The preceramic exhibits a ceramic yield of 20.55 wt % at 450 °C and even shows almost no weight loss up to 800 °C. Most of the mass loss during thermal degradation probably originates from various changes such as the cleavage of terminal methyl groups and the evolution of nitrogen and hydrogen. Thus, the pyrolytic conversion of a-CSEB polymer into the desired amorphous ceramic is achieved. The relatively low decomposition temperature and low yield are a consequence of oxygen atoms introduced via the molecular precursor that survive the polymerization procedure and its low dimensional (linear carbon chain) structure which are essential for high ceramic yields. This might be verified by introducing some high UV sensitivity groups without oxygen atoms and high carbon contents into the pre-ceramic polymers. It is necessary to improve the cross-linking density of the polymer and, therefore, its ceramic yield during the polymer-to-ceramic conversion [22].

### 2.5. Characterization of the a-CSEB Ceramic

The as-obtained ceramics are heated up to 1100 °C in a N_2_ atmosphere, then measured by FTIR (Figure 2c and Table 2). The infrared spectrum shows a strong and sharp absorption band centered at 3145 cm^−1^ which is characteristic of free carbon C−C bonds. BN stretching absorptions appear at 1402 cm^−1^ while the band at 1105 cm^−1^ is assigned to Si−O deformations. All this indicates a kind of ceramic containing the elements Si, B, C, O and N.

**Figure 7:**
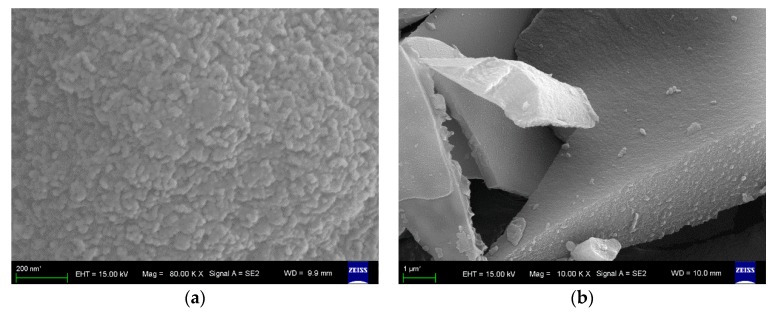
Scanning electron micrograph of the ceramic derived from a-CSEB. (**a**) Surface microstructure of as-obtained ceramic; (**b**) The overall morphology of ceramic.

Scanning electron micrograph (SEM) images of the ceramic after the pyrolysis process at 1100 °C are shown in Figure 7, which show that the surface of ceramic material is almost smooth and dense, since it has formed a protective film consisting of SiO_2_ during the sintering process. Indeed, the as-obtained ceramic material has a large number of pores. This is because during the pyrolysis process, various small molecules are formed and escape. Moreover, the non-crystallinity of the silicon boron carbonitride could be predicted by the irregular surface and morphology of the ceramic grains with sharp edges.

**Figure 8:**
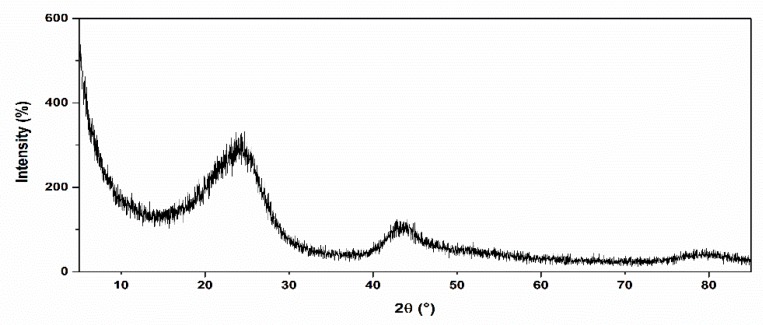
XRD powder patterns of a-CSEB derived ceramics after heating up to 1100 °C in a helium flow.

Furthermore, the amorphous character of the as-obtained ceramic is analyzed by an X-ray powder diffraction (XRD, Figure 8) which only gives three broad diffuse peaks around 25°, 43° and 77°, which are attributable to the h-BN (002), h-BN (100) and h-BN (110) surface, respectively. However, all the broad diffuse peaks were not especially sharp or obvious, revealing the ceramic remains essentially amorphous, even at very high temperature.

## 3. Experimental Section

### 3.1. Materials

All reactions were carried out in a nitrogen atmosphere. Boron trichloride (BCl_3_, Dongguan Ruihe Chemical Corporation, Dongguan, China) was condensed at −30 °C and was stored in a refrigerator. Chlorodimethylvinylsilane (CH_2_CH(CH_3_)_2_SiCl, Energy Chemical Corporation, Tianjin, China), triethylsilane (Et_3_SiH, Energy Chemical Corporation), hexamethyldisilazane (HMDS, HWRK Chemical Corporation, Beijing, China), and 2-hydroxyethyl acrylate (CH_2_CHCOOCH_2_CH_2_OH, Energy Chemical Corporation) were distillated before use.

### 3.2. Synthesis of Single-source Ceramic Precursors

#### 3.2.1. Synthesis of Chloro(dimethyl)silyldichloroborylethane (CSDE) 

**Scheme 1:**
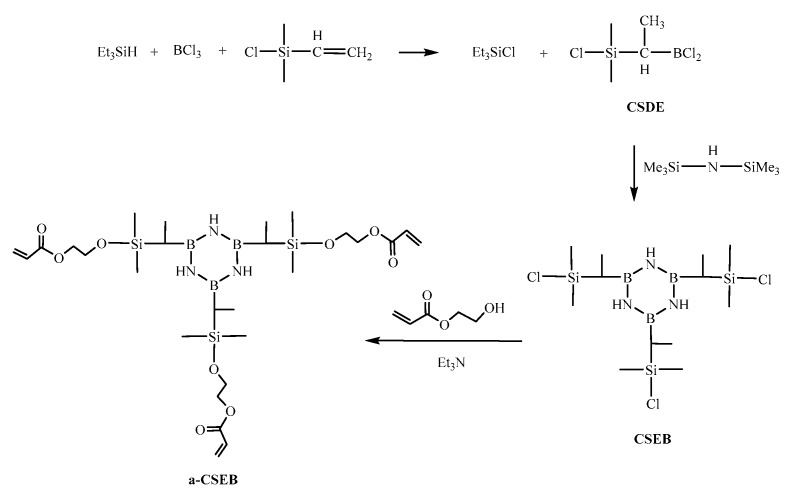
Synthesis of a-CSEB.

Typically, the molar ratio of BCl_3_, CH_2_CH(CH_3_)_2_SiCl, Et_3_SiH was 1:1:1. BCl_3_ (5.9 g, 0.05 mol) and of CH_2_CH(CH_3_)_2_SiCl (6.0 g) were introduced into a pre-cooled 250 mL round-bottomed-flask. Et_3_SiH (5.8 g) was slowly added dropwise to the solution under a vigorous stirring at −65 °C with a syringe, and then the temperature of the solution was increased to room temperature. The excess BCl_3_ were removed at room temperature under vacuum for 1 h. The product was a transparent liquid which was extremely sensitive to moisture and air. The synthesis route of CSDE is shown in Scheme 1 [18].

#### 3.2.2. Synthesis of B,B′,B′′-chloro(dimethyl)silylethylborazine (CSEB)

Theoretically, the molar ratio of HMDS and CSDE was 1:1, HMDS (8.1 g) was added dropwise to CSDE with stirring. The temperature of the mixture was kept below −10 °C until the addition was complete. Then, the reaction mixture was heated to 25 °C and was stirred for 4 h. Finally, byproducts and low molecular oligomers were removed under a vacuum for about 1 h, and a yellow liquid was obtained, yielding 67% of CSEB.

#### 3.2.3. Synthesis of B,B′,B′′-(dimethyl)ethylacrylatesilyloxyethylborazine (a-CSEB)

The molar ratio of CSEB and CH_2_CHCOOCH_2_CH_2_OH was 1:3. CSEB (5.0 g)was dissolved in tetrahydrofuran (120 mL), and Et_3_N (5.1 g) was introduced as an acid neutralizing reagent. CH_2_CHCOOCH_2_CH_2_OH (5.8 g) was added dropwise into the mixture solution at 0 °C using a dropping funnel. The reaction mixture was then allowed to warm up to room temperature and maintained at ambient temperature for 72 h with stirring. Then the solvent and byproduct were removed by filtration, washing, centrifugation, and column separation [12]. A transparent yellow liquid was thus obtained, yielding 94% of a-CSEB.

### 3.3. Ultraviolet Light Polymerization

Benzoin (5%) was added into a-CSEB as a photo-initiator, and when fully dissolved, the mixture was put under UV light irradiation for 5 min (light intensity was 110 mW/cm^2^), and a yellow transparent solid was obtained, yielding 92% of a-CSEB polymer.

### 3.4. Pyrolysis

The UV cured materials were put into a tubular furnace (GSL-1800X, Kejing Materials Technology Corporation, Hefei, China). Subsequently, the tubular furnace was heated to 1100 °C and held for 4 h (heating rate 6 °C/min) under a flowing nitrogen atmosphere. Then, the tubular furnace was cooled in ambient air to room temperature. A black ceramic was obtained. The preceramic exhibits a ceramic yield of 20 wt%.

### 3.5. Characterization

Fourier transform infrared (FTIR) spectra of samples were obtained with a Tensor 27 spectrometer (Bruker, Karlsruhe, Germany) in KBr pellets. ^1^H-, ^13^C-, ^11^B- and ^29^Si- nuclear magnetic resonance (NMR) spectra were obtained in CDCl_3_ using a Bruker Advance apparatus operating at 600 MHz. The UV polymerization was studied by Photo-Differential Scanning Calorimetry (Photo-DSC) using a UV-DSC8000 instrument (Mettler Toledo, Zurich, Switzerland). When the sample was irradiated at room temperature using 110 mW/cm^2^ light, efficient curing was observed. Real-time FTIR was used to study the UV curing process in real time. UV radiation from a point lamp (FUTANSI, Shanghai, China) was introduced into the FTIR spectrometer sample chamber. The light guide was inclined about 15 degrees so that it did not block the path of the IR beam. A mechanical shutter was provided to control the exposure time. The radiation intensity was controlled by changing the distance between sample and lamp and was held constant at 110 mW/cm^2^. Each spectrum was acquired after exposure under the UV light was 10 s. The spectra were obtained one by one in order to analyze the UV curing process in real time. Thermogravimetric analysis (TGA) was conducted with a Q600SDT instrument (TA, Newcastle County, DE, USA). The sample was heated under nitrogen atmosphere at 10 °C/min from 25 to 800 °C. Pyrolysis products were revealed by scanning electron microscopy (SEM) with a S-3000N instrument (Hitachi, Ibaraki-ken, Japan). X-ray diffraction (XRD) experiments were performed on the ceramic using a XRD-7000 instrument, with Cu-Ka radiation (Shimadzu Ltd., Kyoto, Japan).

## 4. Conclusions

In this study, a borazine-type UV photo-induced polymerization of a ceramic precursor was examined. The six-membered borazine rings in the a-CSEB molecule precursors became part of the preceramic polymer via crosslinking of the precursor. As a special structural feature, the precursor molecules have excellent UV-curing propertiies, with a double-bond conversion of around 92.75% after exposure under UV irradiation for 40 s or longer. In addition, the decomposition temperature and temperature of the maximum weight loss of the UV-cured polyorganosilazane are about 100 and 450 °C, and the residue weight at 800 °C is about 20.55 wt %. The relatively low decomposition temperature and low yield is the consequence of the introduction of oxygen atoms via the molecular precursor and its low dimensional (linear carbon chain) structure. The scanning electron micrography and X-ray powder diffraction results indicated the ceramic retained its amorphous nature even at 1100 °C. In further research, grafting other photo-sensitive functional groups onto the backbone of preceramic polymer will be a promising direction to follow

## Figures and Tables

**Table 1:** NMR spectroscopic data of the a-CSEB molecule.

Nucleus	Observed Multiplicity	δ/ppm	Assignment
^1^H	Doublet	5.72/6.33	–CH=CH_2_
^1^H	Triplet	6.02	–CH=CH_2_
^1^H	Quartet	1.75	–CH–CH_3_
^1^H	Doublet	1.18	–CH–CH_3_
^1^H	Singlet	4.0	NH
^1^H	Triplet	4.13	Si–O–CH_2_–CH_2_–O
^1^H	Triplet	3.71	Si–O–CH_2_–CH_2_–O
^13^C	Singlet	129.03	–CH=CH_2_
^13^C	Singlet	131.16	–CH=CH_2_
^13^C	Singlet	166.53	C=O
^13^C	Singlet	26.19	–CH–CH_3_
^13^C	Singlet	68.36	–CH–CH_3_
^13^C	Singlet	61.54	–CH_2_–CH_2_–O–
^13^C	Singlet	66.35	–O–CH_2_–CH_2_–
^11^B	Singlet	30.00	
^29^Si	Singlet	19.93	

**Table 2:** FTIR data of the a-CSEB molecule, a-CSEB polymer and a-CSEB ceramic.

Vibrational Mode	a-CSEB Molecule Wave Number/cm^−1^	a-CSEB Polymer Wave Number/cm^−1^	a-CSEB Ceramic Wave Number/cm^−1^
ν (NH)	3494	3494	
ν (C–C)			3145
ν (CH_3_)	2961	2961	
ν (CH)	2875	2875	
ν (C=O)	1735	1735	
ν (C=C)	1640/1410		
ν_as_ (B_3_N_3_)	1462	1462	1402
δ_as_ (CH_3_)	1340	1340	
δ_s_ (CH_3_)	1250	1250	
ν (BC)	1133	1133	
δ (NH)	1069	1069	
ν (SiO)	1002	1002	1105
δ (C=C)	979		
ν_as_ (B_3_N_3_)	840	840	810
ν (SiC)	746	746	
γ(NH)	610	610	

**Table 3:** NMR spectroscopic data of the a-CSEB polymer.

Nucleus	Observed Multiplicity	δ/ppm	Assignment
^1^H	Quartet	1.75	–CH–CH_3_
^1^H	Doublet	1.18	–CH–CH_3_
^1^H	Singlet	3.79	NH
^1^H	Triplet	4.11	Si–O–CH_2_–CH_2_–O
^1^H	Triplet	3.58	Si–O–CH_2_–CH_2_–O
^1^H	Triplet	2.40	–CH_2_–CH_2_–CH_2_–CH_2_–
^1^H	Triplet	1.70	–CH_2_–CH_2_–CH_2_–CH_2_–
^13^C	Singlet	175.02	C=O
^13^C	Singlet	26.19	–CH–CH_3_
^13^C	Singlet	66.49	–CH–CH_3_
^13^C	Singlet	60.51	–CH_2_–CH_2_–O–
^13^C	Singlet	65.75	–O–CH_2_–CH_2_–
^13^C	Singlet	31.92	–CH_2_–CH_2_–CH_2_–CH_2_–
^13^C	Singlet	22.68	–CH_2_–CH_2_–CH_2_–CH_2_–
^11^B	Singlet	33.36	
^29^Si	Singlet	21.09	
